# Comparative genomics using long-read sequencing identifies nearly identical TAL effector regions in two *Xanthomonas oryzae* pv. *Oryzae* isolates collected from the basmati rice-growing region of Pakistan

**DOI:** 10.3389/fmicb.2025.1560969

**Published:** 2025-06-03

**Authors:** Khansa Ejaz, Muhammad Zakria, Peiqi Zhang, Jose Huguet Tapia, Muhammad Arif, Frank White, Sumera Yasmin

**Affiliations:** ^1^Soil and Environmental Biotechnology Division, National Institute for Biotechnology and Genetic Engineering College, Pakistan Institute for Engineering and Applied Sciences (NIBGE-C, PIEAS), Faisalabad, Punjab, Pakistan; ^2^National Agricultural Research Centre, Crop Diseases Research Institute, Islamabad, Pakistan; ^3^Department of Plant Pathology, University of Florida, Gainesville, FL, United States; ^4^Agricultural Biotechnology Division, National Institute for Biotechnology and Genetic Engineering College, Pakistan Institute for Engineering and Applied Sciences (NIBGE-C, PIEAS), Faisalabad, Punjab, Pakistan

**Keywords:** rice, bacterial leaf blight, TAL effectors, RVDs, Oxford Nanopore Sequencing, type III secretion system

## Abstract

The emergence of hostile and novel plant pathogenic strains poses a serious threat to global food security, which renders the strategies for disease management in modern agriculture ineffective. Preventing the consequences of these emerging phytopathogens requires accurate genetic information about the pathogen population to formulate effective management strategies. Bacterial leaf blight (BLB), caused by *Xanthomonas oryzae* pv. *oryzae* (*Xoo*), is the foremost reason for substantial yield losses in rice crops worldwide, especially in Asia. The genetic information regarding the Pakistani *Xoo* population is still unexplored. To bridge this gap, two representative Pakistani *Xoo* isolates, namely PkXoo1 and PkXoo2, were sequenced using long-read Oxford Nanopore Technology (ONT). Both isolates were obtained from the Basmati rice-growing region of Pakistan, with substantially high virulence on certain susceptible rice varieties. The final assembly of PkXoo1 and PkXoo2 yielded a circular chromosome of approximately 4.9 MB with a G + C content of 64%. Genome annotation of both strains revealed the presence of key genes associated with hypersensitivity and virulence in *Xoo*. The AnnoTALE analysis showed that both strains contained 18 transcription activator-like (TAL) effectors, three of which were predicted to be pseudoTALes. A phylogenomic analysis grouped PkXoo1 and PkXoo2 with strains belonging to India and Thailand, placing them far apart from other major Asian *Xoo* strains. The present study revealed significant findings about the conservation of repeat variable di-residues (RVDs) in major TAL effectors and the utility of high-throughput sequencing technologies for TAL effector analysis and pathogen tracking. The complete genome sequence of *Xoo* isolates from Pakistan will enhance sequence resources for the global comparison of *Xoo* diversity across the region. This information is also of great significance for launching effective and durable breeding programs.

## 1 Introduction

Outbreaks of diseases caused by *Xanthomonas* have been reported worldwide across multiple hosts (Quezado Duval et al., 2004). The most studied and widely prevalent xanthomonads are pathovars of *Xanthomonas oryzae,* which cause bacterial leaf blight (BLB) and bacterial leaf streak (BLS) in rice (Timilsina et al., 2020). Bacterial Leaf Blight (BLB) is a destructive rice disease caused by *Xanthomonas oryzae* pv. *oryzae* (*Xoo*). It affects almost all rice-growing regions worldwide and is considered a serious yield-limiting factor in rice production. *Xoo* is a vascular pathogen that penetrates the host plant via hydathodes and wounds (Niño-Liu et al., 2006).

Key aspects of the mechanism underlying BLB disease have been elucidated, providing an efficient roadmap for breeding resistance. To successfully colonize the host and induce disease symptoms, *Xoo* secretes a series of transcription activator-like (TAL) effectors into the host through the type III secretion system (T3SS) (Boch and Bonas, 2010; Jacques et al., 2016). TAL effectors translocate to the host nucleus, where they bind to effector-specific DNA bases in the promoter region of specific genes and trigger the transcription of either susceptibility (S) or executor (E) host genes (Bogdanove et al., 2010). They bind to their target genes in a continuous, code-like manner, which is specified by the central domain or central repeat region (CRR) of an individual TAL effector. The CRR consists of nearly identical tandem repeats (typically ranging from 33 to 35 amino acid residues), which form a super-helical structure by aligning around the target DNA. However, the residues at the 12th and 13th positions of all repeats are hypervariable, commonly acknowledged as repeat variable di-residues (RVDs). Each RVD determines an exclusive interaction with a specific, single-base pair of the target DNA sequence. The succeeding RVDs in a TAL effector protein are responsible for binding to a specific DNA sequence located at the promoter region of the targeted gene, called the effector binding elements (EBEs) (Boch et al., 2009; Moscou and Bogdanove, 2009). This code-like mechanism is the key principle of TAL effector-DNA specificity, enabling the determination of TAL effector binding sites in the genome of host plants (Noël et al., 2013). Understanding this mechanism offers exciting novel opportunities for breeders and researchers to develop resistant rice varieties (Hutin et al., 2015).

Generally, activated genes are susceptibility (S) genes that help pathogens colonize the host plant. The Sugars Will Eventually be Exported Transporter (SWEET) family of sugar transporters is considered a well-characterized group of *S* genes, commonly known as *SWEET* genes (Streubel et al., 2013). *SWEET* genes export sucrose into the xylem vessels, which are eventually used by the bacteria for disease progression and symptom development (Carpenter et al., 2020). There are more than 20 *SWEET* genes present in rice, but only 3 of them, *OsSWEET11a* (formerly SWEET11) (Wu et al., 2022), *OsSWEET13,* and *OsSWEET14,* are stated to be upregulated by diverse TAL effectors from different *Xoo* strains (Oliva et al., 2019). These are all members of the Clade III of the *SWEET* gene family. On the other hand, TAL effectors may also activate resistance (R) genes that trigger localized host cell death to restrict host infection, ultimately leading to successful host defense (Bogdanove et al., 2010; Boch et al., 2014).

TAL effector coding genes are assumed to be highly varying and dynamic. Previous population genomic studies have also reported high-degree variations in the genomes of *Xoo* strains. It is crucial to understand the evolution and diversity of TAL effectors, as variations in TAL effector repeats have played a significant role in the production of novel toxins and in breaking plant resistance against pathogens (Erkes et al., 2017; Kaur et al., 2020). Due to the highly repetitive structure and sequence identity among different *tal* genes, assembling these genes within the genome is difficult using conventional sequencing methods, such as first- and second-generation sequencing approaches. Nowadays, the employment of high-throughput, long-read sequencing technologies, such as single-molecule real-time (SMRT) sequencing and Oxford Nanopore Technology (ONT), is proving effective in resolving complex genomic structures, such as repetitive elements. These technologies also enable the identification of the position and organization of bacterial pathogenicity islands encoding virulence effectors, particularly the TAL effector region (Ciuffreda et al., 2021). The comprehensive knowledge gained about the evolution and diversity of the TALome will help assess the durability of resistance genes, which may depend on the conservation of certain TAL effectors. This information is of immense importance for developing more effective and well-defined means to control the pathogen population and for designing effective resistance breeding strategies (Hutin et al., 2015).

Pakistan is the ninth-largest producer and the fourth-largest exporter of premium-quality rice in the world. A substantial amount of revenue is generated from rice exports, contributing significantly to the country’s economy (USDA, 2024). Pakistani deluxe rice, widely known as Basmati, is very popular around the globe. Basmati enjoys an inimitable place in the global market due to its properties such as taste, aroma, and texture (Shahzadi et al., 2018; Akhter and Haider, 2020). In Pakistan, BLB was first reported in 1977 (Mew et al., 1989), and in recent years, it remains one of the major causes of losses in rice crops. This is mainly because information regarding the virulence genes, especially the TAL effector repertoire in the Pakistani *Xoo* population, is still unavailable, leading to a lack of effective control measures. Whole genome-based identification of the TAL effector repertoire in the prevalent Pakistani *Xoo* isolates is not only important to enlarge the geographic coverage of the pathogen’s genomic resources but also to develop information-based, durable resistant cultivars (Ejaz et al., 2022).

The identification of *tal* genes in the Pakistani *Xoo* isolates and their comparison with other *Xoo* strains representing East Asian lineages was the main objective of the present study. This study focused on a comparative genomic analysis to identify the TAL effector repertoire in two *Xoo* strains isolated from the Basmati rice-growing area of Pakistan. The knowledge gained from this study will be helpful in designing more informed host resistance strategies for the development of broad and robust resistance in rice.

## 2 Materials and methods

### 2.1 *Xoo* isolates used

Two *Xoo* isolates were collected from BLB-affected farmer fields in the Basmati rice-growing area of Punjab, Pakistan (31.1704° N, 72.7097° E), between 2017 and 2019. Two representative *Xoo* isolates were selected based on their higher virulence on susceptible rice varieties (IR24 and Super Basmati) and were named PkXoo1 and PkXoo2, with Pk representing Pakistan. PkXoo1 was previously reported in our study (Ejaz et al., 2023), which defined its basic genome features and was used in this study for descriptive comparison. PkXoo1 and PkXoo2 were confirmed as *Xoo* using species-specific (*Xo*3756) and pathovar-specific (*Xoo*80) primers in a multiplex PCR (Lu et al., 2014).

### 2.2 Virulence testing of PkXoo1 and PkXoo2

Preserved *Xoo* colonies stored at −80°C were streaked onto Wakimoto agar plates (Sucrose 2%, Peptone 0.5%, Ca(NO_3_)_2_ 0.05%, FeSO_4_ 0.005%) (Cappuccino and Sherman, 2004). The inoculum was prepared from a single, pure *Xoo* colony in nutrient broth with an optimal density (OD) of 1.0 at 600 nm (OD_600_). Seeds of two susceptible rice varieties, IR24 and Super Basmati, were first sown in plastic pots. The seedlings were transplanted into earthen pots after 15 days. The experiment was conducted in a net house under natural conditions during the rice season (June–October), with 80% relative humidity. Forty-five-day-old rice plants of each differential variety were clip-infected using the scissor dip method (Kauffman and Rao, 1972). A total of 10 rice leaves were inoculated with bacteria, and leaves cut with scissors and dipped in sterilized water were used as the control. Disease symptoms were observed and scored based on the percent Disease Leaf Area (% DLA) 21 days post-inoculation (DPI) using the following formula: %DLA = lesion length/total length × 100.

### 2.3 DNA extraction and long-read sequencing of the *Xoo* isolates using Oxford Nanopore Technology

For nanopore sequencing, bacterial cultures from a single colony were cultivated in nutrient broth at 28 ± 2°C for 48 h at 100 rpm until the OD reached 0.8 at 600 nm. The culture (1 mL) was then harvested in a bio-grade centrifuge tube for 5 min at 13,000 rpm. DNA was extracted using the Thermo Fisher Scientific GeneJet Genomic DNA Purification Kit (Massachusetts, United States) following the manufacturer’s protocol. The samples were sequenced using ONT (Oxford, United Kingdom). The extracted DNA was cleaned up with 0.6 volume of AMPure XP beads (Beckman-Coulter, California, United States) to attain the recommended concentration of DNA for Nanopore library preparation and to remove small DNA fragments. This step was also repeated during library preparation after fragmentation. DNA quantity and quality were measured using a NanoDrop^Tm^ One/OneC Microvolume UV–Vis Spectrophotometer (Thermo Fisher Scientific, Massachusetts, United States) to determine the A260/280 and A260/230 ratios, as well as a Qubit 4 Fluorometer (Thermo Fisher Scientific, Massachusetts, United States). For long-read sequencing of each strain, 2–3 μg of genomic DNA was used to prepare the sequencing library. The library was prepared using the PCR-free Rapid Sequencing Kit (RAD004) and Rapid Barcoding Kit (RBK004) following the manufacturer’s protocol (ONT, Oxford, United Kingdom). Oxford Nanopore sequencing was performed at the Department of Plant Pathology, University of Florida, FL, United States. A total of 12 μL of the prepared library from each sample was loaded onto the flow cell according to the manufacturer’s protocol. Multiplexed (barcoded) samples were sequenced using a MinION Flow Cell (R9.4.1) (ONT, United Kingdom) for 72 h. Single samples were also sequenced using a Flongle Flow Cell (R 9.4.1) for 24 h. Sequencing progress was monitored in real-time through the MinKNOW (version: 21.06.13 and 22.05.5) graphic user interface with user-defined parameters. The reads obtained were saved automatically in the system, and the data were retrieved in the form of Fast5 files. The Fast5 reads were base-called using Guppy (version 4.4.1) (Wick et al., 2019). Guppy converted the Fast5 reads into FASTQ format.

### 2.4 Genome assembly and post-finishing

The sequenced reads obtained after base-calling were chopped using Porechop^1^ to cleave adapters. Trimmed reads were filtered to remove reads less than 1,000 bp using the Filtlong tool (v0.2.1).^2^ Reads with a quality higher than Q10 and a length greater than 1Kb were *de novo* assembled using Flye (v2.9) (Kolmogorov et al., 2019). The genome assembly derived from Flye was further corrected and refined using Racon (v1.4.3) (Vaser et al., 2017) and Medaka (v1.7.0).^3^ Assembly completeness scores were calculated using Benchmarking Universal Single-Copy Orthologs (BUSCO) (v5.4.3) (Simão et al., 2015) with the xanthomonadales_odb10 dataset, which consists of 1,152 single-copy orthologs. Although the BUSCO matrices of the polished assembly derived after Medaka were quite significant, manual assessment exposed significant problems with homopolymeric nucleotides, resulting in frequent frameshifts and a large number of apparent pseudogenes. The assembly was further rectified using Homopolish (v0.0.1) (Huang et al., 2021). The Circlator (Hunt et al., 2015) was used to fix the start position and to circularize the assembly. Contamination from any other genome was checked using ContEST16S (Lee et al., 2017).

### 2.5 Genome annotation

Genome annotation was performed using Prokka (v1.14.5) (Seemann, 2014) and GeneMarkS-2 + (Lomsadze et al., 2018), as implemented in the NCBI prokaryotic genome annotation pipeline (Tatusova et al., 2016). Gene function and pathway analyses were performed using BLASTP searches against functional databases, including the Clusters of Orthologous Groups (COGs) database (Tatusov et al., 2003), the Gene Ontology (GO),^4^ and the Kyoto Encyclopedia of Genes and Genomes (KEGG) (Kanehisa, 2002).

### 2.6 Prediction of TAL effectors

The TAL effector repertoire of the two *Xoo* genomes was predicted using the “TALE Prediction” tool from the AnnoTALE suite (v1.4.1) (Grau et al., 2016). The final circular assembly was used as input for the prediction of the TAL effector genes. The DNA sequences of the predicted TAL effectors were used as input to identify RVDs. The AnnoTALE class builder with class_definitin_29–07-22 (downloaded in November 2022) was used to assign classes to the TAL effectors based on their RVD patterns. DisTAL, from the QueTAL suite (Perez-Quintero et al., 2015), was used to calculate similarities based on RVDs between the TAL effectors of available reference *Xoo* genomes (PXO99A, PXO86) in the database. The amino acid sequences of the predicted TAL effectors were used as input for DisTAL.

### 2.7 Phylogenetic and comparative genomic analysis of the Pakistani *Xoo* isolates with other *Xoo* strains representing east Asian lineages

A set of *Xanthomonas* genomes was downloaded from the NCBI Assembly database, and complete genomes were aligned using Parsnp (v1.6.2) (Treangen et al., 2014) for phylogenomic analysis. The tree was visualized using FigTree.^5^ Genome-wide pairwise alignments were calculated using Parsnp, and a phylogenetic tree was constructed with RAxML-ng (v1.1.0) (Kozlov et al., 2019) using the GTR = G model and bootstrap with 100 replications. For structural comparison, the complete genomes of PkXoo1 and PkXoo2 were aligned with reference strains (IXO-280, SK2-3, MAFF311018, PXO71, and PXO99A) using progressive Mauve (Darling et al., 2010) with standard preset settings.

### 2.8 Prediction of effector binding elements

After the TAL sequences were extracted, the TALE-NT 2.0 Target Finder tool (Doyle et al., 2012) was used to predict the targets or effector binding elements (EBEs) of some important TAL effectors. The RVD sequence was used as input. Predictions were carried out using the standard preset settings (score cutoff = 3.0; upstream base of the binding site = T). Predictions were made on the promoterome (defined as the 5′ UTR plus 1,000 base pairs upstream of the transcriptional start site of each transcript) for the clade III *SWEET* genes, using the MSU Rice Genome Annotation Project Release 7. The FindM tool from the signal search analysis (SSA) server (Ambrosini et al., 2003) was used with default parameters to determine the distance between the binding site and the TATA box.

## 3 Results

### 3.1 Virulence analysis of the two *Xoo* isolates, PkXoo1 and PkXoo2, collected from the basmati-growing region of Pakistan

The artificially inoculated leaves of the susceptible rice variety Super Basmati showed BLB symptoms, and the percent diseased leaf area (DLA) for both isolates was calculated. On the basis of molecular identification and phenotypic characteristics, both *Xoo* isolates seemed quite similar. The % DLA for both isolates ranged from 50 to 70% on the susceptible varieties, which confirmed the aggressive behavior of both *Xoo* isolates. In the pathotype analysis, PkXoo1 and PkXoo2 showed virulent interactions with rice NILs carrying single BLB-R genes (IRBB3, IRBB4, and IRBB5) (Shah et al., 2025).

### 3.2 Genome characteristics of PkXoo1 and PkXoo2

Two *Xoo* isolates, PkXoo1 and PkXoo2, collected from Basmati-growing areas of Punjab, Pakistan, were selected to compare the *Xoo* genomic information from the Pakistan region, which is currently not available. Oxford Nanopore sequencing with ultra-long reads was used for the complete genome sequencing of the two strains. The total number of bases retrieved after the sequencing of PkXoo1 and PkXoo2 was 636,943,704.00 and 1,315,318,885.00, respectively. The number of reads generated was 193,312.0 and 239,360.0, respectively. Other sequencing statistics are provided in Table 1. The sequenced reads were *de novo* assembled using Flye, resulting in a single circular contig for each strain. Racon and Medaka were used to polish the assembly, and Homopolish was used to give the assembly a final look, which significantly improved the assembly’s BUSCO completeness (Table 2). After circularizing and adjusting the starting site, the final assembly yielded a single circular chromosome consisting of 4,930,582 base pairs (bp) and 4,941,434 bp, with 133X and 274X genome coverage, respectively. No plasmids were found in either *Xoo* strains. The BUSCO results revealed the completeness of the assembly up to 98.8 and 99%, respectively, although no contamination was found in the two genomes, as confirmed by ContEst16. The genome characteristics of PkXoo1 and PkXoo2 and other reference strains are listed in Table 3. The genomes of PkXoo1 and PkXoo2 encoded 749 and 770 pseudogenes, respectively, while in comparison, PXO99A has 759 and MAFF 311018 has 747 pseudogenes (Ochiai et al., 2005; Salzberg et al., 2008). These results revealed that the quality of the ultralong sequencing platform was relatively high.

**Table 1:** Nanoplot results for PkXoo1 and PkXoo2.

Strain name	Total bases	Number of reads	Mean read length	Read N50
PkXoo1	636,943,704.00	193,312.00	3,294.9	7,191.0
PkXoo2	1,315,318,885.00	239,360.00	5,495.1	10,637.0

Sequencing statistics were calculated using Nanoplot (ONT). Read N50 represents “the length of read in a set for which all reads of that length or longer total to 50% of the set’s total size” (Shafin et al., 2020).

**Table 2:** BUSCO results for PkXoo1 and PkXoo2.

Features	PkXoo1	PkXoo2
Complete BUSCO	1,138 (98.8%)	1,138 (98.8%)
Complete and single-copy BUSCO	1,138 (98.8%)	1,137 (98.7%)
Complete and duplicated BUSCO	0 (0.0%)	1 (0.1%)
Fragmented BUSCO	5 (0.4%)	5 (0.3%)
Missing BUSCO	9 (0.8%)	10 (0.9%)
Total BUSCO group searched	1,152	1,152

Benchmarking Universal Single-Copy Orthologs (BUSCO) was used to evaluate the completeness of the genome assembly. The BUSCO results for PkXoo1 and PkXoo2 revealed that both assemblies were 98% complete.

**Table 3:** Genome features of PkXoo1 and PkXoo2 in comparison with reference *Xoo* strains.

Genome Features	PkXoo1 Pakistan	PkXoo2 Pakistan	PXO99A Philippines	MAFF311018 Japan	KACC10331 Korea
Genome Size (bp)	4,930,683	4,941,434	5,238,555	4,940,217	4,941,439
GC Content (%)	63.72	63.69	63.60	63.70	63.69
Genes (Total)	4,587	4,607	4,877	4,939	4,644
CDS	4,384	4,404	4,666	4,438	4,734
tRNA	53	53	54	53	53
ncRNA	144	144	151	147	146
rRNA	2,2,2	2,2,2	2,2,2	2,2,2	2,2,2
TAL effector genes	18	18	19	17	15
Pseudogenes	749	770	759	747	1,013
Plasmid	0	0	0	0	0

CDS, Coding DNA sequence; TAL effector, transcription activator-like effectors.

### 3.3 Genome annotation

Initially, the annotation was performed using the NCBI automated pipeline, and then, Prokka was also used for the annotation of the two *Xoo* isolates. The results revealed the presence of 4,384 and 4,404 protein-encoding genes (CDS) in PkXoo1 and PkXoo2, respectively (Table 3). Like other *Xanthomonas* genomes, both *Xoo* isolates typically encoded pathogenicity/virulence genes, including extracellular hydrolases, extracellular polysaccharides, adhesins, the type 2 secretion system, and type 3 secretion effectors, specifically TAL effectors. Gene functions and pathway analysis were performed using BLASTP against functional databases.

### 3.4 TAL effectors of PkXoo1 and PkXoo2

To interact with the host and promote proliferation, virulence, and dispersal, *Xoo* exploits TAL effectors. The TAL effector repertoires of PkXoo1 and PkXoo2 were annotated using the AnnoTALE suite, which assigned classes based on the RVDs (Grau et al., 2016). The number of predicted TAL effectors and other features is presented in Tables 4, 5. The AnnoTALE analysis revealed the presence of 15 TAL effectors, along with 3 pseudoTALes, in both isolates. Almost two or three pseudoTALes are found alongside the typical *tal* genes in the genome of *Xoo*. TAL effectors encoded by pseudogenes were defined as the presence of an early stop codon, a frameshift mutation, or the insertion of a large fragment that led to the absence of one or all matches to the nuclear localization signal (NLS) consensus or the absence of a string of 35 amino acids with at least 80% sequence similarity to the acidic activation domain sequence (Van Den Ackerveken et al., 1996).

**Table 4:** Predicted TAL effectors in PkXoo1.

Predicted TAL effectors	Class assigned	Position	Strand
PkXoo1-tempTALE1	TalBA	2,373,874–2,377,171	+
PkXoo1-tempTALE2	TalAA	2,378,990–2,381,462	+
PkXoo1-tempTALE3	TalAR	2,383,860–2,387,883	+
PkXoo1-tempTALE4	TalHT	2,996,038–2,999,053	+
PkXoo1-tempTALE5	TalFS	3,001,273–3,003,679	+
PkXoo1-tempTALE6	TalAG	3,229,708–3,233,428	+
PkXoo1-tempTALE7	TalAS	3,234,417–3,238,848	+
PkXoo1-tempTALE8	TalBX	3,239,837–3,243,959	+
PkXoo1-tempTALE9	TalAH	2,356,805–2,360,519	−
PkXoo1-tempTALE10	TalAN	2,351,958–2,355,816	−
PkXoo1-tempTALE11	TalAD	2,213,180–2,217,308	−
PkXoo1-tempTALE12	TalAB	2,206,180–2,209,903	−
PkXoo1-tempTALE13	TalAL	2,201,678–2,205,191	−
PkXoo1-tempTALE14	TalAP	1,283,209–1,286,926	−
PkXoo1-tempTALE15	TalAQ	1,277,795–1,282,220	−
PkXoo1-tempTALE16	TalAO	1,273,395–1,276,806	−
PkXoo1-tempTALE17	TalAE	1,269,398–1,272,407	−
PkXoo1-tempTALE18	TalBM	430,385–434,303	−

**Table 5:** Predicted TAL effectors in PkXoo2.

Predicted TAL effectors	Class assigned	Position	Strand
PkXoo2-tempTALE1	TalHT	1,954,111–1,957,126	+
PkXoo2-tempTALE2	TalFS	1,959,346–1,961,752	+
PkXoo2-tempTALE3	TalAH	2,596,646–2,600,360	+
PkXoo2-tempTALE4	TalAN	2,601,349–2,605,207	+
PkXoo2-tempTALE5	TalAD	2,741,061–2,745,189	+
PkXoo2-tempTALE6	TalAB	2,748,482–2,752,205	+
PkXoo2-tempTALE7	TalJE	2,753,194–2,755,891	+
PkXoo2-tempTALE8	TalAP	3,671,160–3,674,877	+
PkXoo2-tempTALE9	TalAQ	3,675,866–3,680,291	+
PkXoo2-tempTALE10	TalAO	3,681,280–3,684,691	+
PkXoo2-tempTALE11	TalAE	3,685,679–3,688,688	+
PkXoo2-tempTALE12	TalBM	4,527,233–4,531,151	+
PkXoo2-tempTALE13	TalBA	2,573,890–2,577,187	−
PkXoo2-tempTALE14	TalAA	2,569,108–2,571,901	−
PkXoo2-tempTALE15	TalAR	2,563,280–2,567,303	−
PkXoo2-tempTALE16	TalAG	1,728,266–1,731,986	−
PkXoo2-tempTALE17	TalAS	1,722,846–1,727,277	−
PkXoo2-tempTALE18	TalBX	1,717,735–1,721,857	−

**Figure 1:**
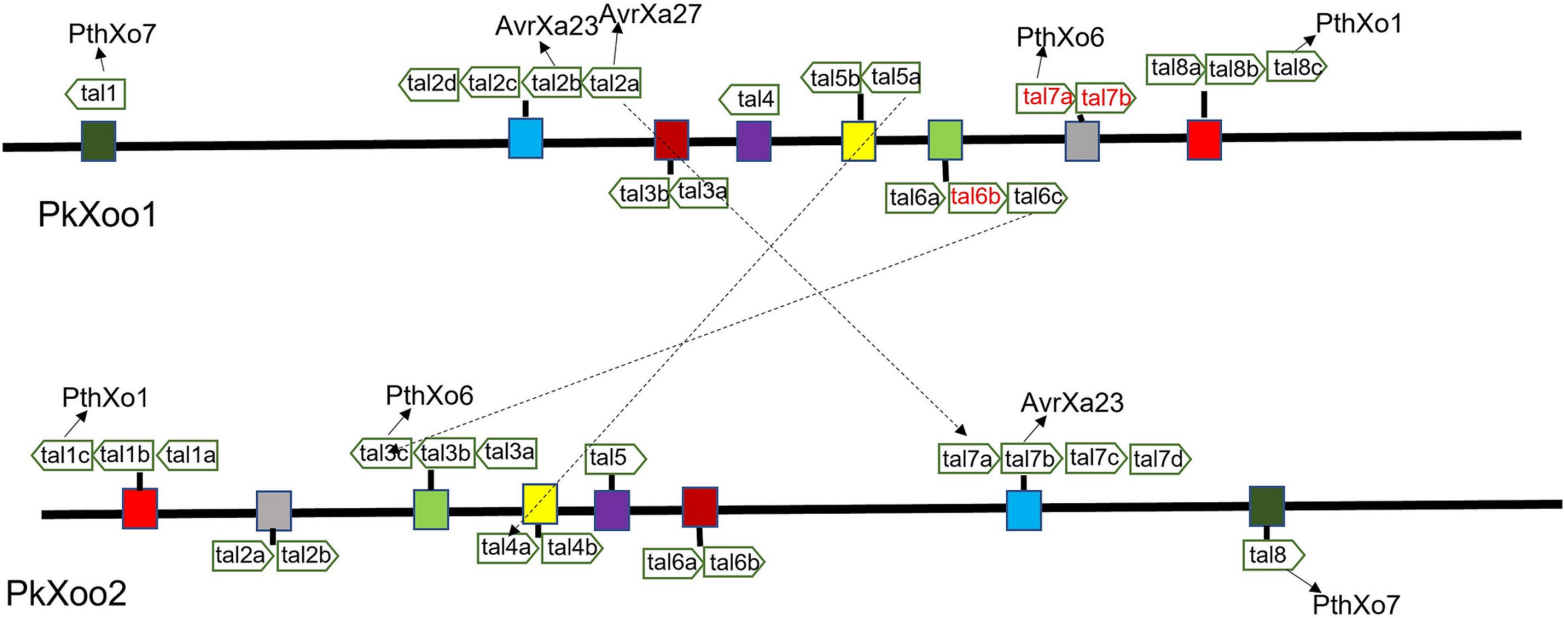
Map of *tal* genes on genomes of PkXoo1 and PkXoo2. The dotted lines represent the TAL effectors with slight differences in RVDs. The same color boxes represent homologous TAL effectors on the genomes of PkXoo1 and PkXoo2.

A total of 15 classic TAL effectors present in both isolates were similar to those in the genomes of other *Xoo* strains, specifically PXO99A and PXO86 (Salzberg et al., 2008). Both strains PkXoo1 and PkXoo2 shared almost the same TAL repertoire, except for the presence of one truncTal in PkXoo2, which was truncated at the 10th repeat. TruncTal is a TAL effector variant with modified N- and C-termini, which may undermine the resistance facilitated by definite non-executor R genes of the host plant (Ji et al., 2016; Read et al., 2016). The shared Tal effectors of both *Xoo* isolates were assigned to the classes TalBM, TalAE, TalAO, TalAQ, TalAP, TalAB, TalAD, TalAN, TalAH, TalBA, TalAA, TalAR, TalHT, TalFS, TalAG, TalAS, and TalBX. Furthermore, truncTal of PkXoo2 was assigned to the class TalJE, whereas its counterpart, which was present in full form in PkXoo1, was assigned to the class TalAL. The *tal* genes were also designated numerically based on the method described by Salzberg et al. (2008). The *tal* genes were positioned at eight loci on the genome. The positions of the *tal* genes in the genome showed rearrangements in both genomes relative to one another (Figure 1).

TAL effector sequences were highly conserved among the *Xoo* isolates, except for the slight difference in the repeats and RVD sequences (Perez-Quintero et al., 2023). PkXoo1 and PkXoo2 had the same RVD pattern for their shared TAL effectors, except for *tal*2a, *tal*5a, and *tal*6c of PkXoo1, which were homologous to *tal*7a, *tal*4a, and *tal*3c of PkXoo2, respectively (Tables 4, 5). These three TAL effectors of both isolates were homologous to PthXo6, *tal*9a, and *ta*l6a in PXO99A. The number of RVDs in the TAL repertoire of both isolates varied from 14 to 27 (Tables 6, 7). *tal*8c of PkXoo1 and *tal*c of PkXoo2 were orthologs of PthXo1 in PXO99A. PthXo1 in PkXoo1 and PkXoo2 had RVD patterns identical to those of PthXo1 in PXO99A.

**Table 6:** Repeat variable di-residue (RVD) pattern of the *Xanthomonas oryzae* pv. *oryzae* strain PkXoo1.

TAL effectors	1	2	3	4	5	6	7	8	9	10	11	12	13	14	15	16	17	18	19	20	21	22	23	24	25	26	27	Orthologs in PXO strains
Tal1	NI	NG	NI	NI	N*	HD	HD	HD	N*	NI	NI	NI	NG	HD	HG	NN	NS	NN	HD	HD	NG	N*						PthXo7 (PXO99A)
Tal2d	NI	NN	NI	HG	HG	HD	NG	*	HD	HG	HD	HD	HD	NG														
Tal2c	NI	NN	N*	NG	NS	NN	NN	NN	NI	NN	NI	NG	HD	HD	NI	HG	N*											Tal2a (PXO86)
Tal2b	HD	HD	NN	NN	NI	NG	HD	S*	HG	HD	NG	N*	NG	HD	HD	N*	NI	NI	NN	HD	HI	ND	HD	NG	NN	HG	N*	
Tal2a	HD	HD	HD	NG	N*	NN	HD	HD	N*	NI	NI	NN	HD	HI	ND	HD	NN	NI	NG	NG								
Tal3b	NI	NN	N*	NG	NS	NN	HD	N*	NN	NN	NI	NN	HD	NG	HD	HD	HD	NG										PthXo6 (PXO99A)
Tal3a	NI	HG	NI	NI	NI	NN	HD	NS	NN	NS	NN	HD	NN	NI	HD	NN	NI	NG	HD	NG								Tal5b (PXO86)
Tal4	NN	HD	NS	NG	HD	NN	N*	NI	HD	NS	HD	NN	HD	NN	HD	NN	NN	NN	NN	NN	NN	NN	HD	NG				Tal5a (PXO86)
Tal5b	NI	HG	NI	HG	NI	NI	NI	HD	NN	HD	NS	NG	SS	HD	NI	NI	NN	NI	NN	NI	NG							Tal6a (PXO99A)
Tal5a	NI	N*	NI	NS	NN	NG	NN	NS	N*	NS	NN	NS	N*	NI	HG	HD	NI	HD	HD	NG								Tal4b (PXO86)
Tal6a	NI	NS	HD	HG	NS	NN	HD	H*	NG	NN	NN	HD	HD	NG	HD	NG												Tal8e (PXO86)
Tal6b	*	*	NS	HG	HG	HD	NS	NG	HD	NN	NG	HG	NG	HD	HG	HD	HD	NI	NN	NG								Tal8a (PXO99A)
Tal6c	NI	H*	NI	NN	NN	NN	NN	NN	HD	NI	HD	HG	HD	NI	N*	NS	NI	NI	HG	HD	NS	NS	NG					
Tal7a	NS	HD	NG	Ng	HG	NG	HD	HD	NG	HD	NN	HD	NG	HD	NI	NI	NI	N*										Tal9a (PXO99A)
Tal7b	NS	NG	NG	NG	NG	HD	HD	NN	NG	HD	NG	NG	HD	HD	HD	H*												AvrXa23 (PXO99A)
Tal8a	NI	NG	NN	NG	NK	NG	NI	NN	NI	NN	NI	NN	NS	NG	NS	NN	NI	N*	NS	NG								AvrXa27 (PXO99A)
Tal8b	NI	HG	NI	NN	NS	HD	NN	HD	HG	HD	NI	NI	NN	NI	HD	HD	HD	HG	NN	NN	HD	NS	NN	HD	N*	NS	N*	Tal8d (PXO86)
Tal8c	NN	HD	NI	NG	HD	NG	N*	HD	HD	NI	NG	NG	NI	HD	NG	NN	NG	NI	NI	NI	NI	N*	NS	N*				PthXo1 (PXO99A)

Residues in red indicate differences in the RVDs from their homolog gene, and * specifies any missing amino acid residue in a certain RVD.

**Table 7:** Repeat variable di-residue (RVD) pattern of the *Xanthomonas oryzae* pv. *oryzae* strain PkXoo2.

TAL effectors	1	2	3	4	5	6	7	8	9	10	11	12	13	14	15	16	17	18	19	20	21	22	23	24	25	26	27	Orthologs in PXO strains
Tal1c	NN	HD	NI	NG	HD	NG	N*	HD	HD	NI	NG	NG	NI	HD	NG	NN	NG	NI	NI	NI	NI	N*	NS	N*				PthXo1 (PXO99a)
Tal1b	NI	HG	NI	NN	NS	HD	NN	HD	HG	HD	NI	NI	NN	NI	HD	HD	HD	HG	NN	NN	HD	NS	NN	HD	N*	NS	N*	
Tal1a	NI	NG	NN	NG	NK	NG	NI	NN	NI	NN	NI	NN	NS	NG	NS	NN	NI	N*	NS	NG								
Tal2a	NS	HD	NG	ng	HG	NS	HG	HD	NG	HD	NN	HD	NG	HD	NI	NI	NI	N*										
Tal2b	NS	NG	NG	NG	NG	HD	HD	NN	NG	HD	NG	NG	HD	HD	HD	H*												
Tal3c	NI	H*	NI	NN	NN	NN	NN	NN	HD	NI	NS	HG	HD	NI	N*	NS	NI	NI	HG	HD	NS	NS	NG					PthXo6 (PXO99a)
Tal3b	*	*	ns	HG	HG	HD	NS	NG	HD	NN	NG	HG	NG	HD	HG	HD	HD	NI	NN	NG								
Tal3a	NI	NS	HD	HG	NS	NN	HD	H*	NG	NN	NN	HD	HD	NG	HD	NG												
Tal4a	NI	N*	NI	NS	NN	NG	NN	NS	N*	NS	NN	NS	N*	HG	HG	HD	NI	HD	HD	NG								Tal6a (PXO99a)
Tal4b	NI	HG	NI	HG	NI	NI	NI	HD	NN	HD	NS	NG	SS	HD	NI	NI	NN	NI	NN	NI	NG							
Tal5	NN	HD	NS	NG	HD	NN	N*	NI	HD	NS	HD	NN	HD	NN	HD	NN	NN	NN	NN	NN	NN	NN	HD	NG				
Tal6a	NI	HG	NI	NI	NI	NN	HD	NS	NN	NS	NN	HD	NN	NI	HD	NN	NI	NG	HD	NG								Tal8a (PXO99a)
Tal6b	NI	NN	N*	NG	NS	NN	HD	N*	NN	NG																		iTALE
Tal7a	HD	HD	HD	NG	N*	NN	HD	HD	N*	NI	NI	NN	HD	HI	ND	HD	NI	HD	NG	NG								Tal9a (PXO99a)
Tal7b	HD	HD	NN	NN	NI	NG	HD	S*	HG	HD	NG	N*	NG	HD	HD	N*	NI	NI	NN	HD	HI	ND	HD	NG	NN	HG	N*	AvrXa23 (PXO99a)
Tal7c	NI	NN	N*	NG	NS	NN	NN	NN	NI	NN	NI	NG	HD	HD	NI	HG	N*											AvrXa27 (PXO99a)
Tal7d	NI	NN	NI	HG	HG	HD	NG	*	HD	HG	HD	HD	HD	NG														
Tal8	NI	NG	NI	NI	N*	HD	HD	HD	N*	NI	NI	NG	HD	HG	NN	NS	NN	HD	HD	NG	N*							PthXo7 (PXO99a)

Residues in red indicate differences in the RVDs from their homolog gene, while * specifies any missing amino acid residue in a certain RVD.

### 3.5 Phylogenetic and genome comparison

**Figure 2:**
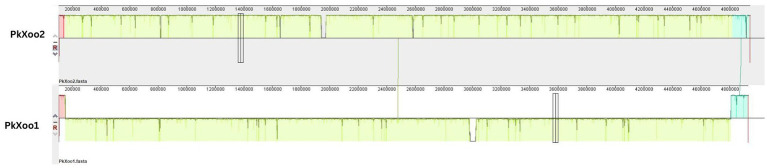
Comparison of the genome structure of PkXoo1 and PkXoo2. The colors represent locally colinear blocks (LCBs), which are regions that show no rearrangement across all the compared genome sequences. Synteny is displayed by a line that links orthologous LCBs. LCBs found on the bottom part represent regions that are found in reverse orientation.

Phylogeny based on whole genome alignment showed that both Pakistani isolates shared a strong relationship with isolates from neighboring Asian countries—*Xoo* IX-280 from India and *Xoo* SK2-3 from Thailand. To determine the relationship between the two isolates, a pairwise genome comparison was conducted using Mauve. The results showed that the chromosomes of PkXoo1 and PkXoo2 somehow exhibited synteny (Figure 2), but PkXoo1 showed genomic rearrangements relative to PkXoo2. The genome alignment results indicated that the genomes of the two Pakistani isolates were conserved in gene content and allelic sites. Although the two isolates were genetically identical, genomic rearrangements were observed (Figure 2).

**Figure 3:**
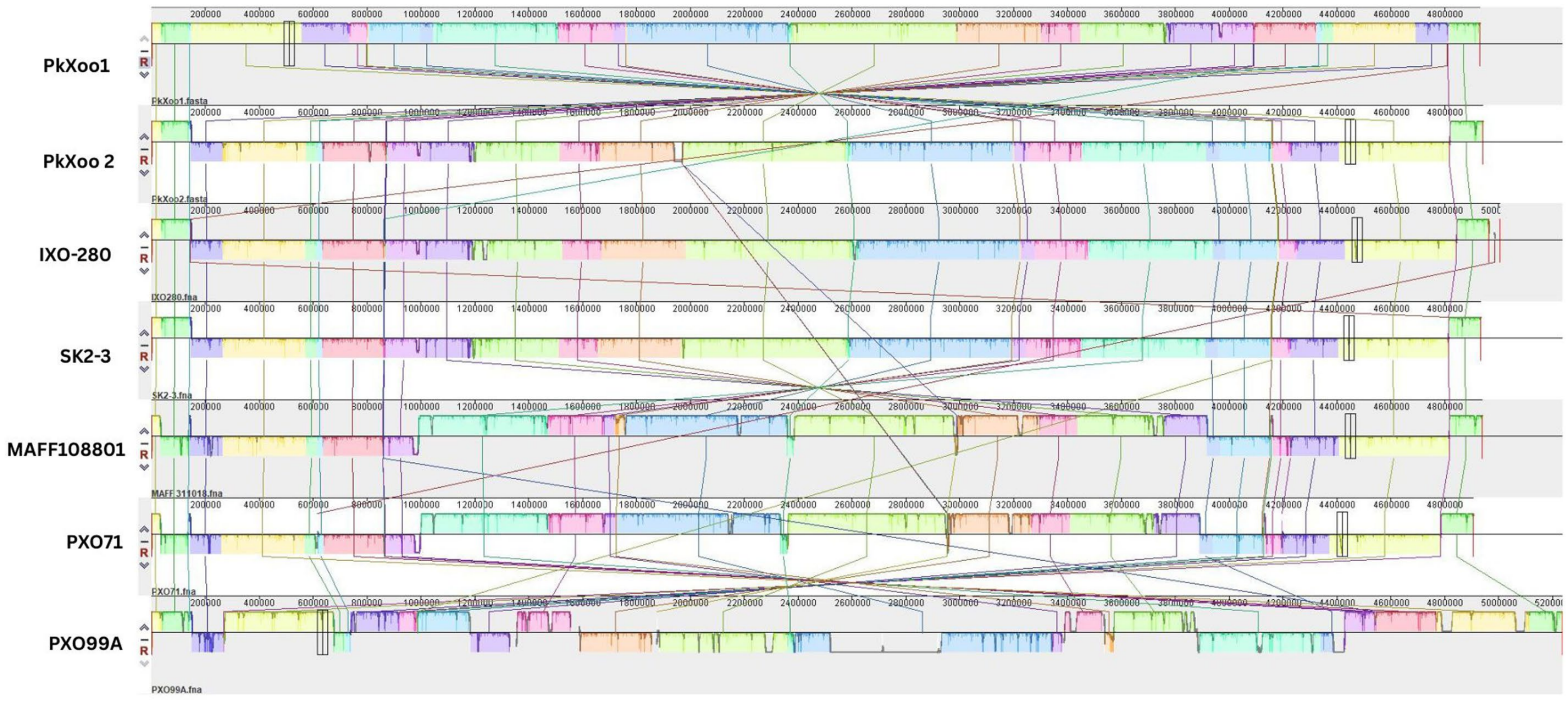
Pairwise comparisons of the chromosome structure of PkXoo1 and PkXoo2 using available complete genome sequences of reference *Xoo* strains from other Asian countries.

To compare the genome structures of PkXoo1 and PkXoo2 with the genomes of the other *Xoo* strains representing East Asian lineages (Quibod et al., 2016), a pairwise genome comparison was performed using Mauve. The alignment showed that the genome structure of PkXoo2 was similar to that of the Indian strains IX-280 and SK2-3 from Thailand. While PkXoo1 showed genomic rearrangements, its genome structure was similar to that of other closely related *Xoo* strains (Figure 3).

### 3.6 TAL effector EBE prediction

In the present study, we assessed the target EBEs of important TAL effectors present in the Pakistani *Xoo* isolates using an *in silico* target prediction tool. TalBX (homologous to PthXo1) and TalBM (homologous to PthXo7) were found in both Pakistani isolates. Strains that can cope with *xa5-*mediated resistance showed the presence of either TalBX, which corresponds to the PthXo1 TAL effector, or TalBM, which corresponds to the PthXo7 TAL effector of *Xoo.* For PthXo1 or TalBX, EBEs were predicted to be present in the promoter region upstream of locus LOC_Os08g42350.1, which corresponds to the *OsSWEET11* gene. Similarly, for TalBM, EBEs were predicted in the promoter region upstream of locus LOC_Os01g73890, which corresponds to the *OsTFIIAγ1* gene.

## 4 Discussion

BLB is a primary threat to rice crops, caused by *Xanthomonas oryzae* pv. *oryzae* (*Xoo*). Resistance to the BLB pathogen includes a durable, race-specific element conferred either by disease resistance genes or by engineered variations in the effector-binding sites of susceptibility genes. To date, 46 resistance (*Xa*) genes have been discovered and widely incorporated into BLB-resistant varieties (Tian et al., 2014).

Pakistan, being one of the largest rice-producing countries, exports fine-quality rice all over the world. However, to date, BLB remains one of the major constraints to achieving significant rice production in Pakistan. Outbreaks of BLB have occurred repeatedly in Pakistan, specifically in the Kallar belt, which is famous for producing high-quality Basmati rice. Recent surveys have indicated a high occurrence of BLB in Punjab province (~30–55%) (Waheed et al., 2009; Ullah et al., 2022). The knowledge regarding the virulence factors of the Pakistani *Xoo* isolates is still unexplored, which hinders breeding efforts to overcome this disease (Ejaz et al., 2022). In the present study, we sequenced two Pakistani *Xoo* isolates, PkXoo1 and PkXoo2, collected from the Kaller belt region of Punjab province, Pakistan, using the Oxford Nanopore MinION sequencer. The emergence of cost-effective, long-read, next-generation sequencing technologies has facilitated the study of whole genomes and the identification of identical/nearly identical repetitive elements and genes encoding proteins, such as TAL effectors. The potential and power of the Oxford MinION sequencer in the investigation of the *Xanthomonas* genus, including *Xoo* strains, have also been reported in previous studies (Bansal et al., 2018; Kaur et al., 2019; Fernandes et al., 2021; Erkes et al., 2023). This study can provide valuable insights into the diversity of TAL effectors in Pakistani *Xoo* and assist in the discovery of key TAL effectors essential for virulence.

Both *Xoo* isolates had a homolog of PthXo1, namely *tal*8c in PkXoo1 and *tal*1c in PkXoo2. Both shared the same RVD pattern as PthXo1 in PXO99. Previously, Carpenter et al. (2020) reported a difference of one RVD in the PthXo1 ortholog of IX-280 and SK2-3 compared to PXO99. PthXo1 is the prominent virulent determinant that binds directly to the *SWEET11a* promoter in rice, delivering the mechanism behind gene-for-gene recessive resistance and susceptibility (Chen et al., 2010; Römer et al., 2010). The function of PthXo1 is interrupted in rice by the recessive blight resistance gene *xa*13 (Salzberg et al., 2008).

The Pakistani *Xoo* isolates, PkXoo1 and PkXoo2, did not encompass any other major TAL effectors predicted to trigger the expression of any other *SWEET* gene from clade III. Remarkably, an identical copy of PthXo7, the PXO99A TAL effector that targets TFIIAγ1, was also present in both strains (*tal*1 in PkXoo1 and *tal*8 in PkXoo2). PkXoo1 and PkXoo2 also had orthologs of the PXO99A TAL effector PthXo6, homologous to *tal*3b in PkXoo1 and *tal*3c in PkXoo2. PthXo6 triggers the expression of the bZIP transcription factor TFX1. AvrXa23 and AvrXa27, which are considered avirulent determinants and trigger the response of *X*a23 and *Xa*27 resistance genes in rice, were also present in both Pakistani *Xoo* isolates. The RVD pattern of AvrXa23 is different from its ortholog in PXO99A but is identical to the AvrXa23D present in PXO61, PXO71, Sk2-3, and IX-280, as reported recently by Xu et al. (2022). Carpenter et al. (2020) also reported the harmony of IX-280 and SK2-3 with *xa*5.

The phylogenomic analysis and genome comparison indicated that the Pakistani isolates PkXoo1 and PkXoo2 are closely related but show rearrangements and inversions compared to one another. Further analysis revealed the presence of a large number of transposases and insertion sequences in the genome of both *Xoo* isolates, which are likely to contribute to genome plasticity. Such a high number of insertion sequences within the genome facilitates chromosomal recombination, horizontal gene transfer, and gene duplication (Vandecraen et al., 2017). Previously, a relatively large number of insertion sequences were reported in *Xoo,* as these mobile elements were involved in race differentiation. Insertion elements can also lead to the loss of gene function, and virulence can develop from the inactivation of avirulence genes (Ochiai et al., 2005; Goettelmann et al., 2023).

Both *Xoo* isolates PkXoo1 and PkXoo2 were highly identical to the Indian *Xoo* strain IX-280 and a *Xoo* strain from Thailand, SK2-3. Both strains belong to the young and relatively high clonal lineage L-1, which is predominant in India (Midha et al., 2017; Carpenter et al., 2020). The genome structure of PkXoo2, IX-280, and SK2-3 is comparable to each other, despite the three strains belonging to different regions. In contrast, the *Xoo* strains from the Philippines, PXO86, PXO71, and PXO99A, have experienced genomic rearrangements relative to one another, although they belong to the same region (Carpenter et al., 2020).

Although geographically separated, PkXoo2 shares a striking genomic similarity with IX-280 and SK2-3. This has prompted us to investigate the relationship between these isolates and other *Xoo* strains. The clonality of PkXoo2 with IX-280 and SK2-3 suggested that immigration played a significant role in the evolution of local *Xoo* populations (Carpenter et al., 2020). The results highlight the necessity of developing local varieties with distinct or even better stacked resistance genes for rapid deployment.

The relatedness of the Pakistani isolates to IX-280 and SK2-3 suggests that migration events, such as the exchange of seeds and the import and export of agricultural commodities, may be involved in the evolution of local *Xoo* populations (Carpenter et al., 2020). The presence of an ortholog of PthXo1 in these strains underlines the effectiveness of a complete genome sequence study using long-read sequencing technologies and TAL effector analysis-based surveillance to comprehend the breakdown of resistance genes. The results also emphasize the need for the development of local cultivars with diverse single or, preferably, stacked BLB resistance genes for the expeditious deployment of BLB-resistant cultivars.

## 5 Conclusion

The present study reports the TAL effector repertoire in two *Xanthomonas oryzae* pv. *oryzae* (*Xoo*) isolates from Pakistan. The whole genome sequencing using long-read sequencing technology provided a deep understanding of diversity, genome plasticity, and geographical relationships of the *Xoo* isolates. The findings of this study will be helpful to enhance sequence resources for the global comparison of pathogen diversity across the region. The presence of 17 distinct TAL effectors in both *Xoo* isolates unravels their virulence potential. Phylogenetic analysis surprisingly unveiled the relatedness of the Pakistani *Xoo* isolates with the Indian and Thai strains, suggesting the potential role of migration events in shaping the pathogen population. In the future, to uncover the population structure and TALome diversity in Pakistan, we aim to focus on sequencing more *Xoo* isolates. This will not only be helpful for disease surveillance in real time but will lead to sustainable and targeted disease management in rice crops.

## Data Availability

The complete genome sequence of PkXoo1 and PkXoo2 has been submitted to NCBI Gen Bank in Bio-project PRJNA861287 under accession number CP101721.2 and CP140752, respectively.

## Footnotes

^1^ https://github.com/rrwick/Porechop

^2^ https://github.com/rrwick/Filtlong

^3^ https://github.com/nanoporetech/medaka

^4^ https://geneontology.org/docs/go-annotations/

^5^ http://tree.bio.ed.ac.uk/software/figtree/
